# Water-Mediated Isomerization in the Stepwise Hydration of the Nitrobenzene Radical Cation with 1–6 Water Molecules

**DOI:** 10.1021/acs.jpca.5c08557

**Published:** 2026-03-23

**Authors:** Zachary A. Christensen, John P. Saunier, Kyle A. Mason, Ka Un Lao, M. Samy El-Shall

**Affiliations:** Department of Chemistry, 6889 Virginia Commonwealth University, Richmond, Virginia 23284-2006, United States

## Abstract

The gas-phase stepwise hydration of the nitrobenzene radical cation with 1–6 water molecules has been investigated by means of ion mobility mass spectrometry and density functional theory (DFT) calculations. The stepwise binding energies (Δ*H*°_*n*–1,*n*_) were determined by equilibrium measurements for C_6_H_5_NO_2_^·+^(H_2_O)_*n*_ with *n* = 1–3 as 13.4, 12.7, and 10.8 kcal/mol, respectively. DFT calculations indicate that the first three hydration steps are facilitated by noncovalent interactions of the associating water molecules with calculated enthalpy changes of 13.1, 12.8, and 10.7 kcal/mol, respectively, in excellent agreement with the experimentally determined Δ*H*° values. However, the fourth hydration step involves irreversible addition of water to the C_6_H_5_NO_2_^·+^(H_2_O)_3_ cluster, resulting in the formation of a covalently bonded ion hydrated by three water molecules [C_6_H_7_NO_3_^·+^(H_2_O)_3_]. DFT calculations indicate that the covalently bonded ion C_6_H_7_NO_3_^·+^ is formed by the insertion of a water molecule between the ortho carbon and the oxygen atom of the nitro group of C_6_H_5_NO_2_^·+^, resulting in a nitronic phenol-type structure hydrated by three water molecules [(OH)­C_6_H_5_(NOOH)^·+^(H_2_O)_3_]. The fifth and sixth hydration steps were demonstrated to be in equilibrium reversible associations with experimental −Δ*H*° values of 11.7 and 12.1 kcal/mol, respectively, in good agreement with the calculated values of 11.9 and 11.4 kcal/mol corresponding to the formation of (OH)­C_6_H_5_(NOOH)^·+^(H_2_O)_4_ and (OH)­C_6_H_5_(NOOH)^·+^(H_2_O)_5_ clusters, respectively, of the hydrated nitronic phenol-type radical cation (OH)­C_6_H_5_(NOOH)^·+^. The observed transition constitutes the first example of water-mediated isomerization of the noncovalent microhydrated nitrobenzene, leading to a favorable pathway for the formation of the covalently bonded nitronic phenol-type radical cation. The formation of the nitronic phenol-type radical cation has significant implications for the production of gas-phase reactive hydrated organic nitrates (RONO_2_)^·+^(H_2_O)_*n*_ in atmospheric organic aerosols, which could contribute to air quality in a variety of environments.

## Graphical abstract

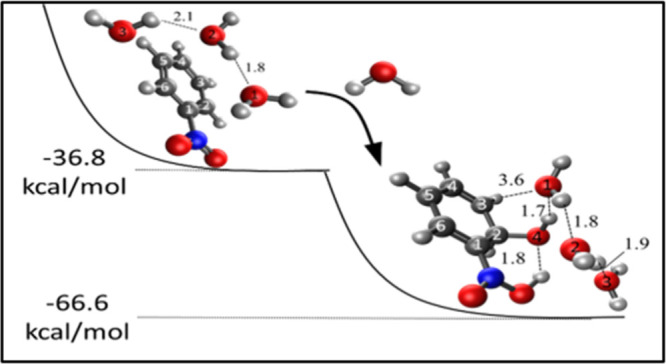

## Introduction

Investigation of the stepwise hydration of gas-phase organic ions allows for a detailed understanding of interactions taking place in ion solvation. This can include the formation of inner or external solvation shells of neutral solvent molecules onto an ion. These interactions are critical in many fields such as gas-phase ion chemistry, ion-induced nucleation, radiation chemistry, astrochemistry, and astrobiology.^1–6^ They are likewise important to biological systems, including protein folding, proton transport, membranes, and interfacial or biological water in proteins and bioactive molecules.^1,3^

Previous work investigated the stepwise hydration of various organic radical cations. These include benzene (C_6_H_6_^+·^), cyclobutadiene (C_4_H_4_^+·^), phenylacetylene (C_8_H_6_^+·^), benzonitrile (C_7_H_5_N^+·^), naphthalene (C_10_H_8_^+·^), pyridine (C_5_NH_5_^+·^), and pyrimidine (C_4_N_2_H_4_^+·^).^7–16^ Many of these solvation behaviors involve interactions by unconventional ionic hydrogen bonds (UIHB) of the type CH^δ+^··O, where the ionized hydrocarbon CH groups function as hydrogen donors and the oxygen lone pair electrons of the solvent molecules serve as hydrogen acceptors.^8,10,15,17,18^ Another interesting aspect of these interactions is found in the change in reaction pathway that can be observed with higher numbers of solvent molecules. In the case of the hydration of pyrimidine, both the radical cation (C_4_N_2_H_4_^+·^) and protonated pyrimidine (C_4_N_2_H_5_^+^) present a dissociative proton transfer pathway to the solvent water cluster.^12^ This only occurs when at least four water molecules are added, which facilitates the thermoneutrality or exothermicity of this alternative reaction pathway.^12^ In the case of the benzonitrile radical cation, the first three additions of water involve the formation of a UIHB between the oxygen of the water molecule and the hydrogens on the ring of the benzonitrile radical cation and a subsequent formation of a hydrogen bonding network among the water molecules.^16^ Upon the fourth addition, though, an alternative reaction pathway becomes available wherein the water facilitates a solvent-assisted isomerization process converting the benzonitrile radical cation (C_6_H_5_CN^+·^) to the distonic ion (^·^C_6_H_4_CNH^+^), which undergoes further hydration as a protonated molecule.^16^

In this paper, we provide a detailed experimental and computational study of the energetics and structures of the hydrated nitrobenzene radical cation C_6_H_5_NO_2_^·+^(H_2_O)_*n*_ with 1–6 water molecules, where two types of interactions may take place with the water molecules. The first is the typical sequential hydration through ion-dipole and hydrogen bonding interactions with the CH^δ+^ groups of the ring (CH^δ+^...OH_2_), resulting in the formation of partially or fully hydrated nitrobenzene radical cations. The second interaction with water could involve a covalent binding motif wherein a hydrogen atom from the water molecule shifts to the nitro group of nitrobenzene and the remaining OH group binds to the ortho carbon of nitrobenzene, thus forming a nitronic phenol-type radical cation that can be stabilized by a strong H-bonding to another water molecule. These covalent additions of hydrogen and oxygen atoms from water certainly involve energy barriers and, therefore, may not take place with a single water molecule. However, by increasing the number of water molecules, the barriers to covalent additions would be reduced, and a transition from the microhydrated nitrobenzene to the hydrated nitronic phenol-type radical cation could take place. The effect of stepwise hydration on the water-mediated isomerization of the nitrobenzene radical cation is the main subject of the present work.

## Experimental and Computational Methods

The experiments were performed using the VCU mass-selected ion mobility (MSIM) system. (Schematic is given in Figure S1, Supporting Information). Details of the instrument can be found in several publications,^16,19,20^ and only a brief description of the experimental procedure is given here. The essential elements of the apparatus are jet and beam chambers coupled to an electron ionization source, a quadrupole mass filter, a drift cell, and a second quadrupole mass spectrometer. Nitrobenzene radical cations (C_6_H_5_NO_2_^·+^) are formed by electron impact ionization (35–40 eV) of the neutral molecules generated using supersonic expansion of 40 psi (2.8 bar) of ultrahigh pure helium seeded with about 1–3% of vapor of the nitrobenzene through a pulsed supersonic nozzle (500 μm) into the vacuum source chamber with 10^–7^ mbar pressure. The radical cations are mass selected using the first quadrupole mass filter and then injected in 100 μs pulses into the drift cell containing a vapor mixture consisting of 25–50 mTorr water in 1.5–2.5 Torr research-grade helium. The injection energies used in the experiments (14–16 eV, laboratory frame) are slightly greater than the minimum energy required to introduce the molecular ions against the outflow of helium escaping the drift cell entrance orifice, yet low enough not to fragment the ions while ensuring complete thermalization. Most of the ion thermalization occurs outside the cell entrance due to collisions with helium atoms and water molecules escaping from the cell entrance orifice. At a cell pressure of 1.4 Torr, the nitrobenzene cation encounters nearly 10^4^ collisions with helium atoms within the typical 1–1.5 ms residence time inside the cell, leading to full thermalization of the ions. The injected ions and hydrated products are separated by scanning a second quadrupole mass filter coaxially positioned downstream from the drift cell. After exiting the quadrupole, ions are detected by an off-axis collision dynode and an electron multiplier. The ion gate pulse simultaneously triggers a multichannel scalar scan to measure the arrival time distributions (ATDs) of the selected ions exiting the cell.

Equilibrium between reactants and products is represented by

$$C_{6}H_{5}{\mathrm{NO}}_{2}^{+\cdot }(H_{2}O{)}_{n-1}+H_{2}O⇌C_{6}H_{5}{\mathrm{NO}}_{2}^{+\cdot }(H_{2}O{)}_{n}$$ 1

when *n* = 1–3 and 5–6. Establishement of equilibrium of the reaction is checked by (1) collection of integrated ion intensity ratios (reactant or product/total ion intensity) as a function of the drift field and (2) collection of the ATDs of the reactant and product ions. Under typical experimental conditions (5 cm drift cell length, 8–12 V, 1–2 Torr He pressure), ATDs range from 0.2 to 1.5 ms. Establishment of equilibrium conditions is supported when the reactant and product ion intensity ratios are constant as a function of reaction time and when there is a significant overlap between the ATDs of the reactant and product ions. Once equilibrium conditions have been established, the equilibrium constant *K*_eq_ can be calculated via the equation

$$K_{\mathrm{eq}}=\frac{I[C_{6}H_{5}{\mathrm{NO}}_{2}^{+\cdot }(H_{2}O{)}_{n}]}{I[C_{6}H_{5}{\mathrm{NO}}_{2}^{+\cdot }(H_{2}O{)}_{n-1}]P_{H_{2}O}}$$ 2

where *I*[C_6_H_5_NO_2_^+·^(H_2_O)_*n*_] and *I*[C_6_H_5_NO_2_^+·^(H_2_O)_*n*−1_] are the integrated ion intensities of the product and reactant, respectively, obtained from the mass spectra, and where *P*_H_2_O_ is the partial pressure of water inside the drift cell in atmospheres. Measurement of *K*_eq_ as a function of temperature yields Δ*H°* and Δ*S°* from the slope and intercept, respectively, of a van’t Hoff plot governed by the equation

$$R\ln(K_{\mathrm{eq}})=-\Delta H^{{}^{\circ}}\left(\frac{1000}{T}\right)+\Delta S^{{}^{\circ}}$$ 3

where *R* is the gas constant (cal mol^–1^ K^–1^), Δ*H°* is the standard change in enthalpy (kcal mol^–1^), Δ*S°* is the standard change in entropy (cal mol^–1^ K^–1^), and *T* is the temperature (K). All values reported were replicated at least three times with an uncertainty of ±1 kcal mol^–1^ and ±2 cal mol^–1^ K^–1^ for Δ*H°* and Δ*S°,* respectively.

Lowest energy structures and associated binding energies were calculated via DFT at the M06–2X level of theory and by employing the 6–311++G** basis set within the Gaussian 16 program suite.^21^ Vibrational frequencies were calculated by allowing for the determination of the zero-point vibrational energy (ZPVE) and the verification of the absence of imaginary frequencies. For the potential energy surface (PES) calculations, initial transition-state structures were automatically and reliably generated using the freezing-string method.^22^ These structures were then optimized as transition states and verified through frequency calculations. All PES calculations and transition-state searches were carried out at the ωB97M-V and the M06-2X levels of theory using the developed version of Q-Chem.^23^

## Results and Discussion

Figure 1 displays the mass spectra obtained following the injection of the nitrobenzene radical cation (C_6_H_5_NO_2_^·+^) into the drift cell containing pure helium buffer gas (1948 mTorr) or a helium–water mixture containing 26–53 mTorr water vapor at the temperature range of 333–194 K. The mass spectrum in pure helium (Figure 1a) shows very minor fragments of the radical cation (C_6_H_5_NO^·+^ and C_6_H_5_^+^) due to the low ion injection energy used in the experiments. In the presence of water, the first hydrated product is observed at 333 K in addition to protonated water clusters H^+^(H_2_O)_*n*_ with *n* ≥ 4 that appear with very low intensity. At lower temperatures, the ion intensity of C_6_H_5_NO_2_^·+^ decreases, and the population of the hydrated clusters C_6_H_5_NO_2_^·+^(H_2_O)_*n*_ increases and shifts to higher *n* values as shown in Figure 1c–f. The hydrated clusters with *n* = 2, 3–4, 5, and 6 are observed at 302, 274, 252, and 230 K, respectively. This general pattern of cluster ion abundance, wherein each higher order addition becomes more favored as the temperature decreases, is typical of a stepwise growth mechanism by means of exothermic reversible processes. However, the simultaneous appearance of the *n* = 3 and 4 clusters at 274 K and the higher abundance of the *n* = 4 cluster at 252 and 233 K suggest an irreversible transition from the *n* = 3 to *n* = 4 cluster. This is also consistent with the observation that no conditions could be achieved wherein the ion intensity of the *n* = 3 cluster is dominant. Instead, the fourth hydration cluster (*n* = 4) shows a relatively high intensity and rapidly supersedes the prevalence of the third hydrated cluster within the temperature range of 275–230 K. At the lowest temperature used in the experiment (194 K, Figure 1g), the water vapor freezes inside the drift cell resulting in almost complete recovery of the ion intensity of C_6_H_5_NO_2_^·+^ and low intensity of the hydrated products C_6_H_5_NO_2_^·+^(H_2_O)_*n*_ with *n* = 1–6.

**Figure 1:**
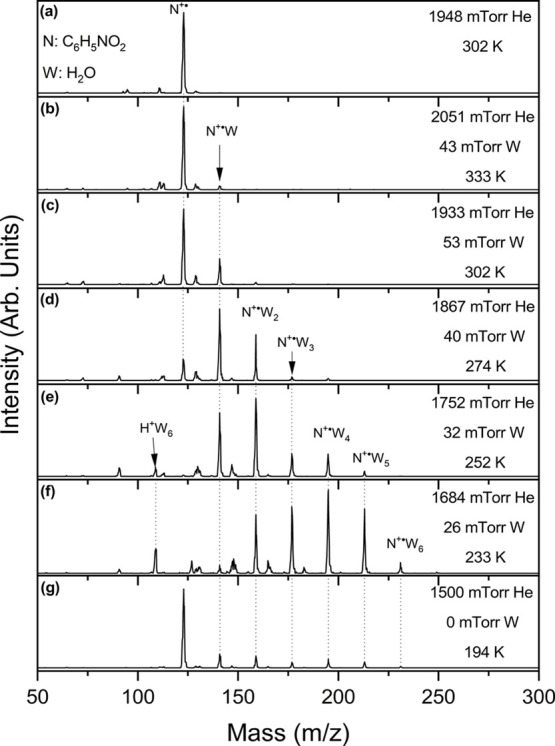
Mass spectra obtained from the injection (15.0 eV) of the nitrobenzene radical cation (N^+·^, C_6_H_5_NO_2_^+·^, *m*/*z* 123) generated by electron ionization into a drift cell starting with 1.95 Torr of He (a) without water (W, H_2_O), and (b–g) with partial pressure of water as indicated at the temperatures of 333, 302, 274, 252, 233, and 194 K, respectively.

To determine whether each of these hydration steps represents an equilibrium process, we analyzed the ATDs of the reactant and product ions. Figure 2a,b show a strong overlap of the ATDs of the species involved in the first and second hydration steps, i.e., C_6_H_5_NO_2_^·+^, C_6_H_5_NO_2_^·+^(H_2_O), and C_6_H_5_NO_2_^·+^(H_2_O)_2_ (N^·+^, N^·+^W, and N^·+^W_2_, respectively, in Figure 2a,b). The strong overlap between the ATDs of the reactant and product ions indicates that these species are coupled in association–dissociation reversible reactions faster than their movements through the drift cell. Moreover, the corresponding ATDs for the species involved in the first (N^·+^W), second (N^·+^W_2_), and third (N^·+^W_3_) hydration steps (Figure 2c) show a strong overlap between these three species at the temperature of 252 K. Similarly, the fifth (N^·+^W_4_/N^·+^W_5_) hydration step is also clearly demonstrated to be in equilibrium as shown by the ATDs in Figure 2d at the temperature of 233 K. On the other hand, the ATDs for the species involved in the fourth hydration process (N^·+^W_3_/N^·+^W_4_) demonstrate a very poor overlap as shown in Figure 2c. This fact, coupled with the unusual enhanced intensity of the ion signal corresponding to the fourth hydration product (Figure 1f), indicates that the fourth hydration process (N^·+^W_3_/N^·+^W_4_) is not at equilibrium. Therefore, the ATDs of the N^·+^W_1–5_ ions shown in Figure 2 suggest the presence of two different populations of hydrated ions represented by the *n* = 1–3 and *n* = 4–6 groups, with no equilibrium that could be established between the two groups. The presence of two different populations of hydrated cluster ions could be explained by a water-mediated isomerization process involving the irreversible addition of water to the C_6_H_5_NO_2_^·+^(H_2_O)_3_ cluster (N^·+^W_3_) and resulting in the formation of a covalently bonded ion C_6_H_7_NO_3_^·+^ (C_6_H_5_NO_2_^·+^ + H_2_O) hydrated by three water molecules [C_6_H_7_NO_3_^·+^(H_2_O)_3_], which can readily add further water molecules reversibly to form the C_6_H_7_NO_3_^·+^(H_2_O)_4_ and the C_6_H_7_NO_3_^·+^(H_2_O)_5_ clusters [equivalent to the C_6_H_5_NO_2_^·+^(H_2_O)_5–6_ clusters] that exhibit no overlapping ATDs with the C_6_H_5_NO_2_^·+^(H_2_O)_1–3_ clusters.

**Figure 2:**
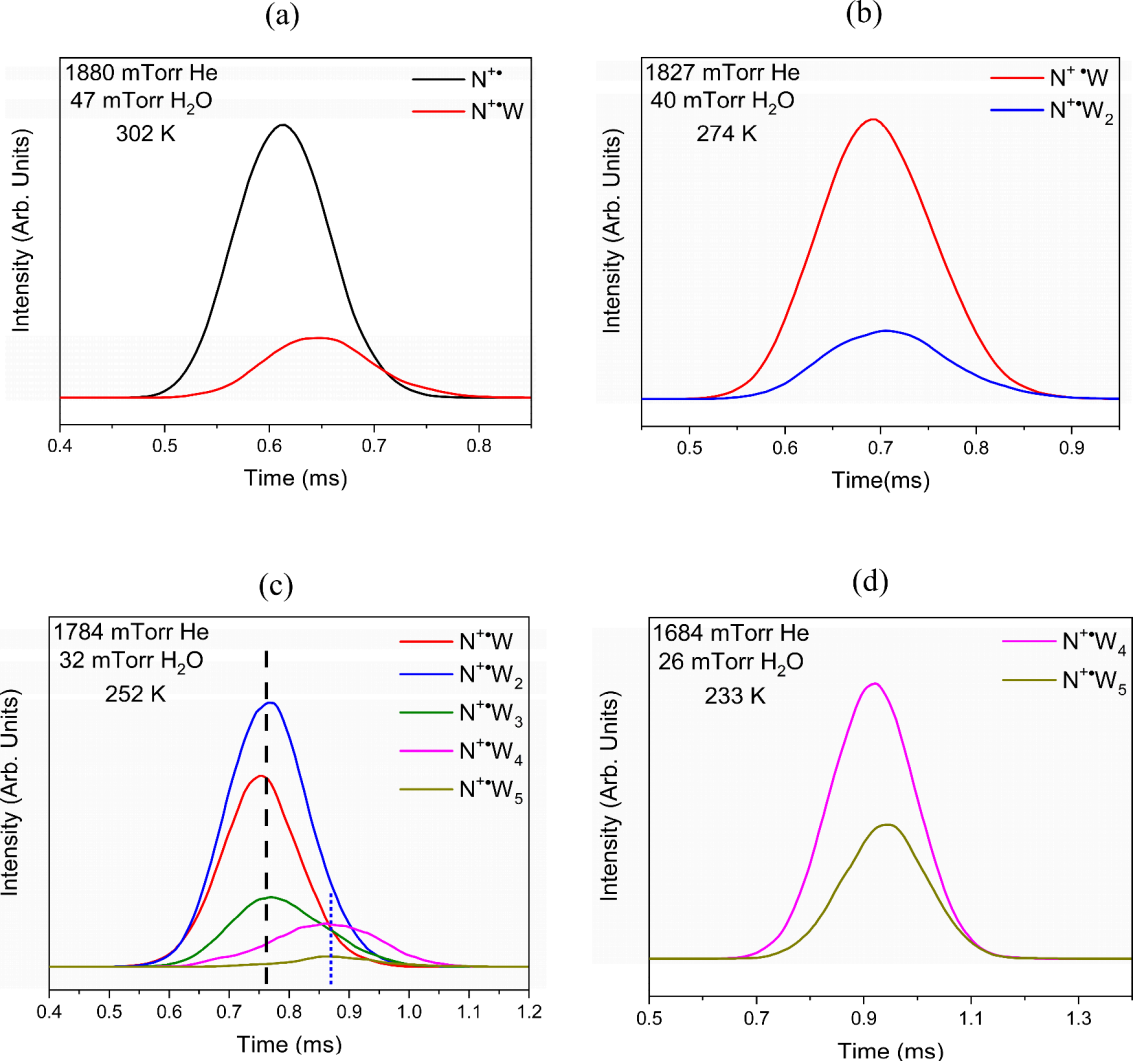
ATDs of the nitrobenzene radical cation (N^+·^) and the N^+·^W_*n*_ hydrated ions corresponding to the C_6_H_5_NO_2_^·+^(H_2_O)_*n*_ clusters with *n* = 1–5. (a) ATDs of N^+·^ and N^+·^W at 302 K; (b) ATDs of N^+·^W and N^+·^W_2_ at 274 K; (c) ATDs of N^+·^W_*n*_ with *n* = 1–5 at 252 K; and (d) ATDs of N^+·^W_4_ and N^+·^W_5_ at 233 K. N^+·^W_4_ and N^+·^W_5_ represent the ATDs of the hydrated covalently bonded ions C_6_H_7_NO_3_^·+^(H_2_O)_3_ and C_6_H_7_NO_3_^·+^(H_2_O)_4_, respectively, as suggested by the experiment.

The normalized ion intensities of the reactant and product ions as a function of reaction time at various temperatures are shown in Figure S2 (Supporting Information). At early reaction times, a constant ratio of the normalized intensity of the product to the reactant ions is not obtained as shown in Figure S2a. However, the ratio of the relative abundance of the product to the reactant ions rapidly approaches a constant value, indicating that equilibrium is most likely established among the ions with overlapping ATD profiles, as shown in Figure S2b–d. Unlike the ions within each of the N^·+^W_1–3_ and N^·+^W_4–6_ populations, which exhibit strong overlapping ATDs and constant ratios of the relative abundance of the product to the reactant ions, no equilibrium could be established between the N^·+^W_3_ and N^·+^W_4_ ions. The forward reaction leading to the formation of N^·+^W_4_ was always too fast to establish equilibrium under all experimental conditions investigated (temperature range = 280–230 K, water’s vapor pressure = 0.01–0.10 Torr, and He pressure = 1–2 Torr). As suggested above, this behavior can be explained by the irreversible addition of water to the C_6_H_5_NO_2_^·+^(H_2_O)_3_ cluster (N^·+^W_3_), resulting in the formation of a covalently bonded ion C_6_H_7_NO_3_^·+^ hydrated by three water molecules [C_6_H_7_NO_3_^·+^(H_2_O)_3_], which is equivalent to the C_6_H_5_NO_2_^·+^(H_2_O)_4_ cluster. The newly formed hydrated covalently bonded ion C_6_H_7_NO_3_^·+^(H_2_O)_3_ can add further water molecules reversibly to form larger hydrated clusters according to the equilibrium reaction 4 as observed:

$$C_{6}H_{7}{\mathrm{NO}}_{3}^{+\cdot }(H_{2}O{)}_{n-1}+H_{2}O⇌C_{6}H_{7}{\mathrm{NO}}_{3}^{+\cdot }(H_{2}O{)}_{n},n=4\text{−}5$$ 4

The equilibrium constants for reaction 1 for the formation of the C_6_H_5_NO_2_^·+^(H_2_O)_*n*_ clusters with *n* = 1–3, and for reaction 4 for the formation of the C_6_H_7_NO_3_^·+^(H_2_O)_*n*_ clusters with *n* = 4–5, determined at different temperatures, yield the van’t Hoff plots displayed in Figure 3a,b, respectively. The resulting −Δ*H*°, −Δ*S*°, and −Δ*G*° (at 250 K) values for the hydration of the nitrobenzene radical cation C_6_H_5_NO_2_^·+^(H_2_O)_*n*_ with *n* = 1–3, and for the hydration of the covalently bonded ion C_6_H_7_NO_3_^·+^(H_2_O)_*n*_ with *n* = 4–5 are summarized in Table 1. The lack of equilibrium in the fourth hydration step involving the irreversible addition of water onto the C_6_H_5_NO_2_^·+^(H_2_O)_3_ cluster could not allow determining the thermochemical parameters for the formation of the hydrated covalently bonded ion C_6_H_7_NO_3_^·+^(H_2_O)_*n*_. Table 1 also includes the calculated −Δ*H*°, −Δ*S*°, and −Δ*G*° (at 250 K) values obtained at the M06–2*X*/6–311++G** level of theory.

**Figure 3:**
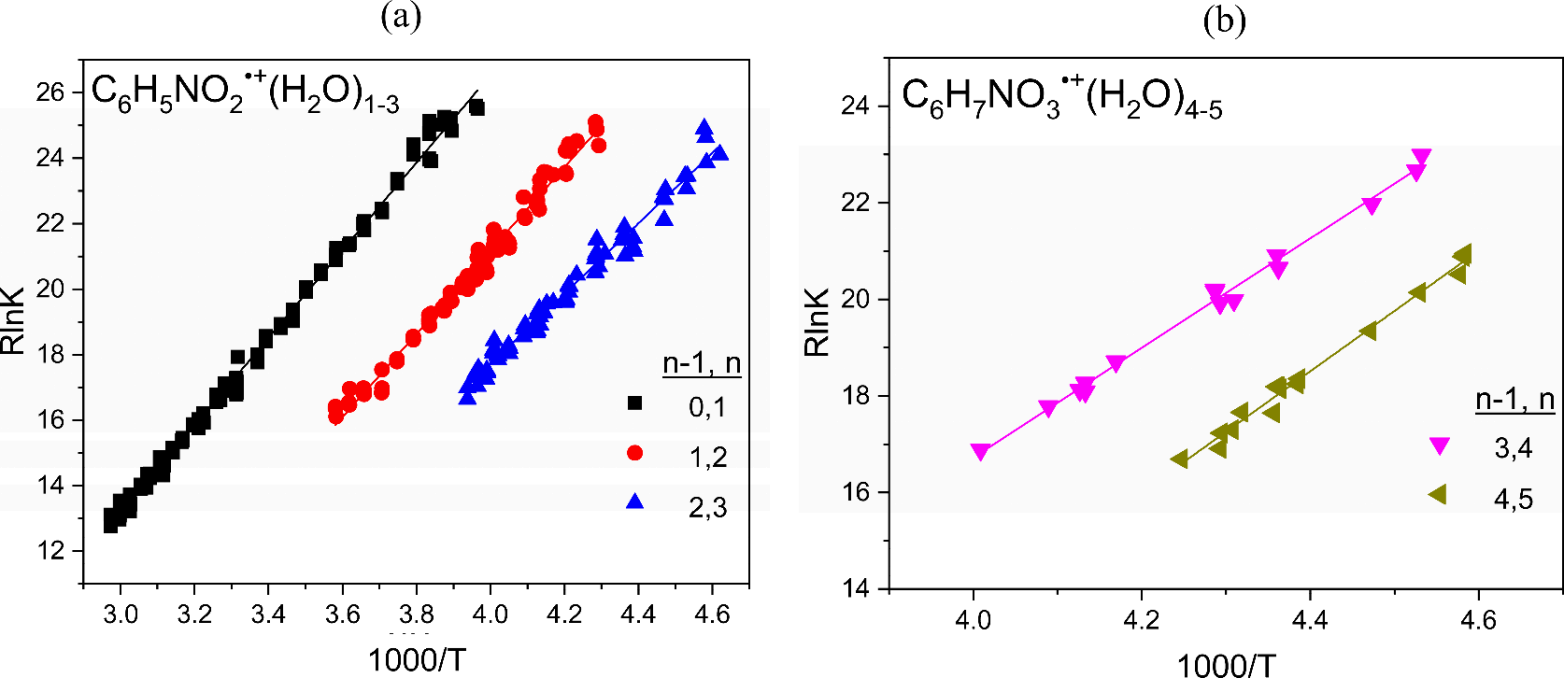
van’t Hoff plots for the temperature dependence of the equilibrium constants for (a) the association of water with the nitrobenzene radical cation C_6_H_5_NO_2_^·+^(H_2_O)_*n*_ with *n* = 1–3 and (b) the association of water with the covalently bonded ion C_6_H_7_NO_3_^·+^(H_2_O)_*n*_ with *n* = 4–5.

**Table 1:** Measured and Calculated Thermochemical Data for the Sequential Hydration of the Nitrobenzene Radical Cation C_6_H_5_NO_2_^·+^(H_2_O)_*n*_ with *n* = 1–3, and the Covalently Bonded Ion C_6_H_7_NO_3_^·+^(H_2_O)_*n*_ with *n* = 4–5.^a^

***n* – 1, *n***	***–*Δ*H°*** (kcal/mol)	***–*Δ*S°*** (cal mol^–1^K^–1^ **)**	***–*Δ*G°*** (250 K) (kcal/mol)
C_6_H_5_NO_2_^·+^(H_2_O)_*n*_			
0, 1	13.4 (13.1)	27.0 (30.8)	6.65 (5.4)
1, 2	12.7 (12.8)	29.7 (30.8)	5.28 (5.1)
2, 3	10.8 (10.7)	25.3 (30.7)	4.48 (3.0)
C_6_H_7_NO_3_^·+^(H_2_O)_*n*_			
3, 4	11.7 (11.9)	29.5 (31.4)	4.33 (4.1)
4, 5	12.1 (11.4)	34.6 (31.9)	3.45 (3.4)

a The calculated values at the M06-2*X*/6-311++G** level are given in brackets.

Figure 4 displays the structures of the lowest energy isomers for the sequential addition of the first three water molecules onto the nitrobenzene radical cation, along with the electrostatic surface potentials (ESP) of these structures mapped on the 0.04 e^–^/bohr^3^ isovalue surfaces obtained from the M06–2*X*/6–311++G** calculations. The lowest energy isomer of the (N^+·^W) cluster, (Figure 4a), has a calculated *–*Δ*H°* of 13.1 kcal/mol, which agrees very well with the experimentally determined *–*Δ*H°* for the first hydration step (13.4 kcal/mol). This structure reflects the charge-dipole interaction wherein the oxygen of the water molecule is associated with the positive charge on the ring, specifically at the substituted nitro group and the ortho position carbon of the ring, as shown in the corresponding ESP map, where areas in dark blue indicate regions of electron deficiency or electropositive regions, while deep red areas represent enhanced electron density. The second and third lowest energy isomers of the (N^+·^W) cluster, shown in Figure S3b,c, (Supporting Information), correspond to structures resulting from the hydrogen bonding between the oxygen of the water molecule and the meta/para (Figure S3b) and ortho/meta (Figure S3c) hydrogens of the ring, respectively. Each of these isomers possesses a calculated *–*Δ*H°* value considerably lower (10.2 and 8.0 kcal/mol) than the experimentally obtained value (13.4 kcal/mol), thus supporting the assignment of isomer (4a) shown in Figure 4a as the most likely structure of the C_6_H_5_NO_2_^·+^(H_2_O) cluster observed in the experiment.

**Figure 4:**
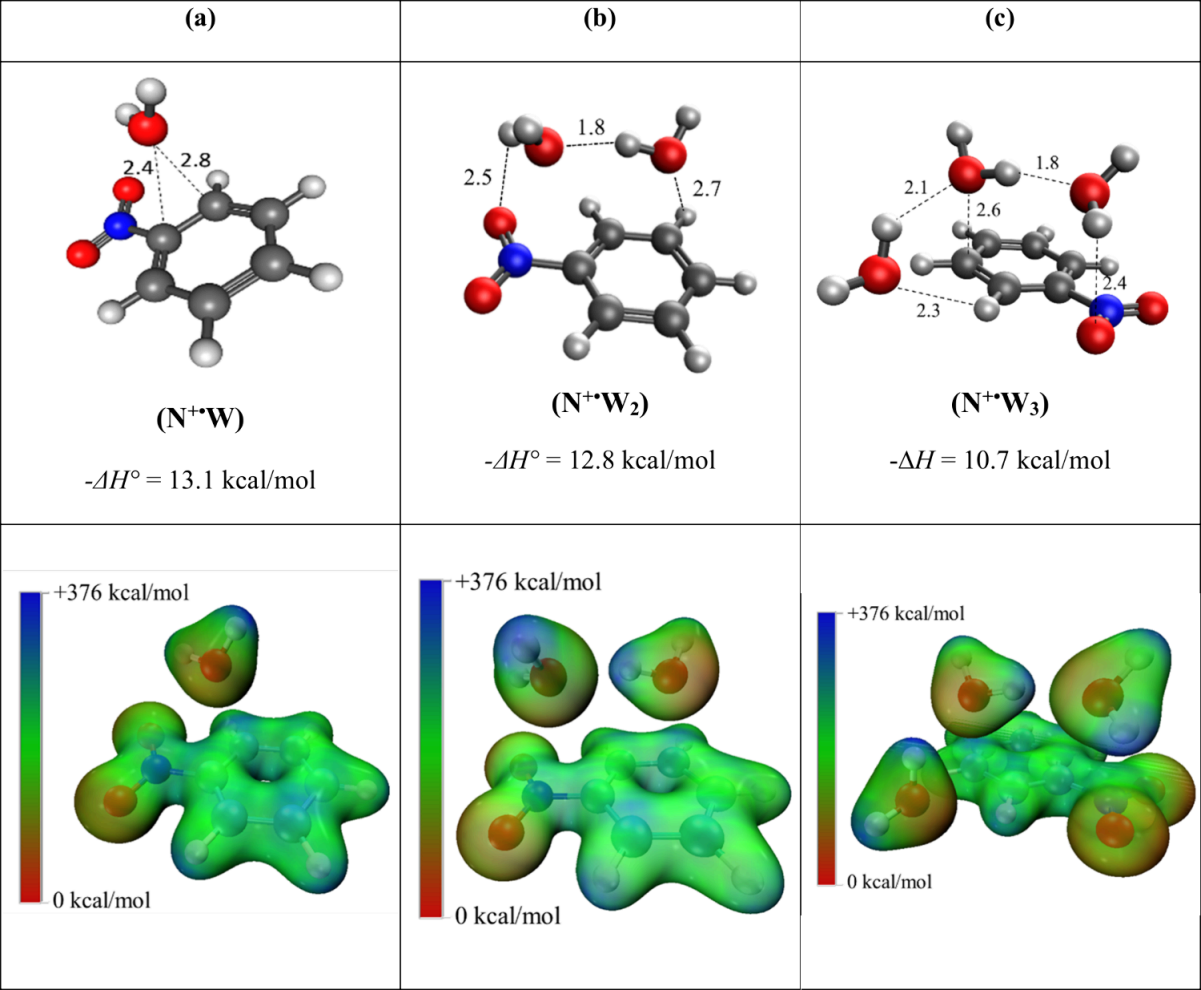
Structures, binding enthalpies (Δ*H°*, kcal/mol), and ESP maps calculated at the M06–2*X*/6–311++G** level of theory for the lowest energy isomers of the C_6_H_5_NO_2_^+·^(H_2_O)_*n*_ clusters with *n* = 1–3. (a) C_6_H_5_NO_2_^+·^(H_2_O) or (N^+·^W) _;_ (b) C_6_H_5_NO_2_^+·^(H_2_O)_2_ or (N^+·^W_2_), and (c) C_6_H_5_NO_2_^+·^(H_2_O)_3_ or (N^+·^W_3_). The distances shown on the dotted lines are in angstroms. Dark blue areas within the ESP maps indicate regions of electron deficiency or electropositive regions, while deep red areas represent enhanced electron density according to the energy scale from +367 to 0 kcal/mol, respectively.

The structures of the calculated lowest energy isomers for the second addition of water onto the nitrobenzene radical cation are displayed in Figure S4 (Supporting Information). The calculated enthalpy change associated with the lowest energy isomer (Figure 4b, −12.8 kcal/mol) is in excellent agreement with the experimentally determined value of −12.7 kcal/mol. Isomer Figure 4b features again an oxygen atom of a water molecule interacting with the nitro group of the nitrobenzene radical cation via charge-dipole interaction as well as engaging in a hydrogen bond (1.8 Å) with the second water molecule. The second and third lowest energy noncovalent isomers reflect interactions with the nitro group and the ortho CH group of the ring (Figure S4b) and an extended H-bonding interaction above the ring (Figure S4c), but both isomers have calculated bonding enthalpy values between 12.4 and 11.8 kcal/mol, a little smaller than the experimentally determined value of 12.7 kcal/mol. Interestingly, this calculation also yielded a covalent association product (Figure 5a), wherein the OH group of one of the water molecules binds covalently to the ortho carbon while the hydrogen is transferred to the nearby oxygen of the nitro group and is connected by a short H-bond (1.7 Å) to the oxygen of the OH group, thus resulting in the formation of a nearly perfect 6-membered ring (N–C–C–O··H–O–N). The newly formed 6-membered ring in the covalent isomer (Figure 5a) is stabilized by an external short H-bond (1.7 Å) with the oxygen atom of the second water molecule. Being a covalent structure, the calculated Δ*H°* associated with the formation of isomer Figure 5a from the reaction of water with isomer Figure 4a, −26.0 kcal/mol, is much lower than the experimentally determined value (−12.7 kcal/mol). Furthermore, the formation of a covalently bonded product would prevent the attainment of reversible equilibrium between the C_6_H_5_NO_2_^·+^(H_2_O) and C_6_H_5_NO_2_^·+^(H_2_O)_2_ ions, contrary to the experimental observation. In addition, the formation of covalent products most likely involves energy barriers which could not be overcome under the high-pressure conditions of the current experiments, where three-body collisional stabilization is very effective in removing any excess energy from the thermalized reactant ions.^8,16–20^ Therefore, it can be concluded that the second hydrated product observed in the experiment is most likely to have the structure of isomer Figure 4b with a calculated −Δ*H°* of 12.8 kcal/mol, in excellent agreement with the experimentally determined value of −12.7 kcal/mol.

**Figure 5:**
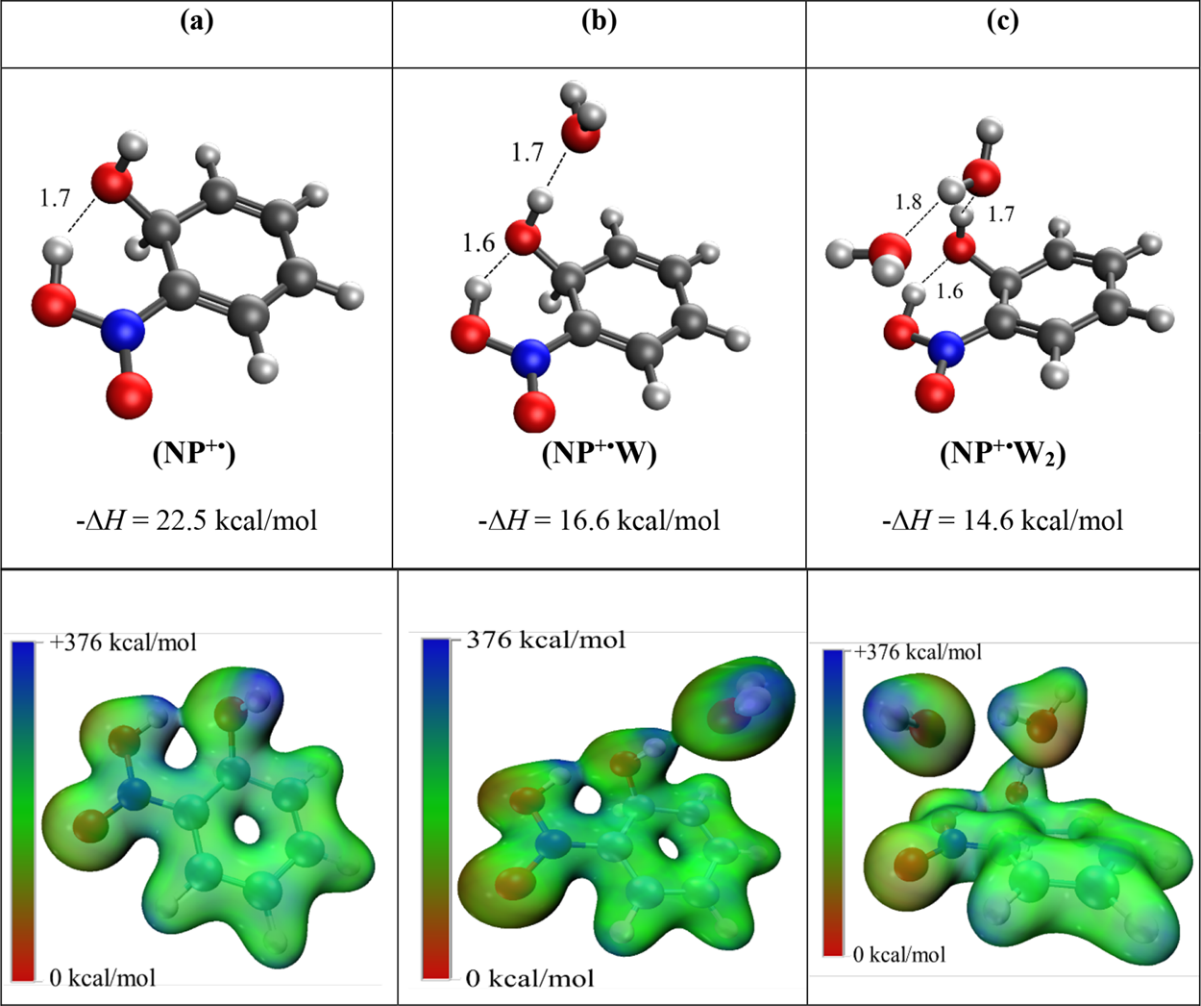
Structures, binding enthalpies (Δ*H*, kcal/mol), and ESP maps calculated at the M06–2*X*/6–311++G** level of theory for the sequential addition of one (b) and two (c) water molecules on the nitrophenol-type radical cation [(HO)­C_6_H_5_(NOOH)]^·+^ (a) formed by the covalent addition of water onto C_6_H_5_NO_2_^+·^. Distances are reported in angstroms. Dark blue areas within the ESP maps indicate regions of electron deficiency or electropositive regions, while deep red areas represent enhanced electron density according to the energy scale from +376 to 0 kcal/mol, respectively.

The calculated structures of the lowest energy noncovalent structures for the third hydration step are displayed in Figure S5 (Supporting Information). Here, the lowest energy noncovalent isomer (Figure 4c) with a calculated enthalpy change of −10.7 kcal/mol is in perfect agreement with the experimentally determined −Δ*H°* of 10.8 kcal/mol. This structure features three water molecules, forming a H-bonded chain above and around the nitrobenzene radical cation. The corresponding ESP map of isomer Figure 4c clearly shows H-bonding interactions among the three water molecules, where the dark blue areas of electron deficiency on the H atoms are close to the red areas, representing enhanced electron density near the oxygen atoms.

Figures 5 and 6 show the structures and the corresponding ESP maps of the first through the sixth sequential addition of water into the covalent binding motif of the nitrobenzene radical cation. These structures all present the binding of the oxygen of the first water molecule to the ortho carbon and hydrogen transfer to the proximal oxygen of the nitro group. This covalent motif is not expected to be present during the early hydration steps (*n* = 1–3) nor is it directly observed experimentally. The irreversible process taking place after multiple noncovalent additions indicates that the presence of a critical number of solvent molecules is required to facilitate an energetic pathway, which makes the covalent addition possible. In this case, it is not until three water molecules are present that the barrier associated with the covalent addition of the fourth water molecule is sufficiently low to permit the addition to take place.

**Figure 6:**
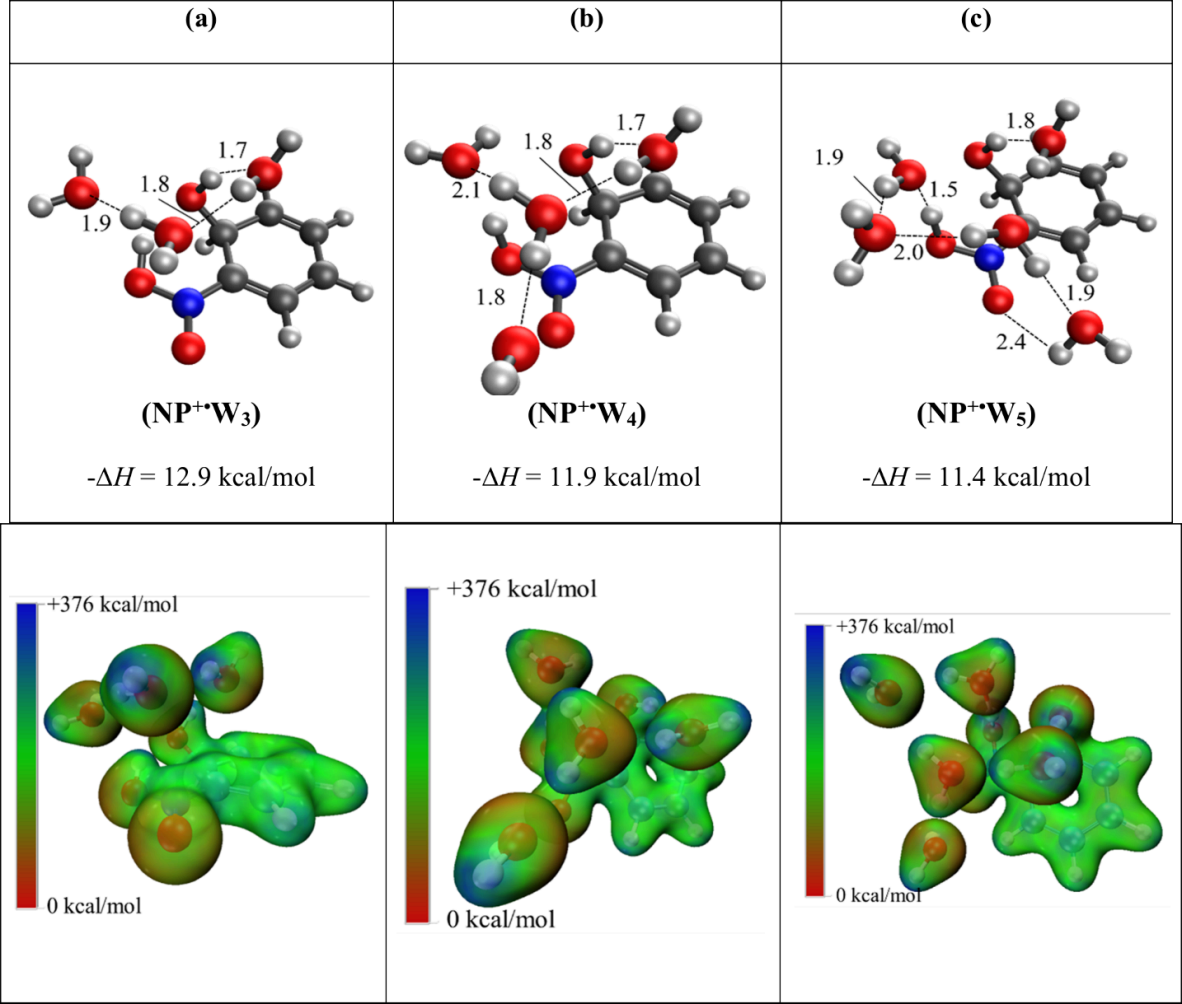
Structures, binding enthalpies (Δ*H*, kcal/mol), and ESP maps calculated at the M06–2*X*/6–311++G** level of theory for the sequential addition of 3, 4, and 5 water molecules on the nitrophenol-type radical cation [(HO)­C_6_H_5_(NOOH)]^·+^ formed by the covalent addition of water onto C_6_H_5_NO_2_^+·^ (shown in Figure 5a). Distances are reported in angstroms. Dark blue areas within the ESP maps indicate regions of electron deficiency or electropositive regions, while deep red areas represent enhanced electron density according to the energy scale from +376 to 0 kcal/mo. respectively.

To investigate the energetics associated with the formation of the covalent structure Figure 5a, we examined the PES for the reaction between water and the nitrobenzene radical cation using the M06–2*X*/6–311++G** level of theory in addition to the ωB97M-V functional, which is known for accurate PES calculations.^22,23^ As shown in Figure 7, the reaction pathway begins with the formation of a noncovalent hydrated complex, [(N^+·^W), structure Figure 4a], corresponding to the association of a single water molecule with the nitrobenzene radical cation. This association is strongly exothermic on the electronic energy surface, with a stabilization of 16.0 – 16.3 kcal/mol relative to the separated reactants. From this hydrated complex, a transition state was identified with a barrier of 2.7–3.0 kcal/mol relative to that of (N^+·^W) [structure Figure 4a]. This transition state involves a concerted proton transfer from water to the N–O group of the nitrobenzene radical cation, accompanied by nucleophilic addition of the hydroxyl group to the ortho carbon, leading to the covalent ion (NP^+·^) [structure Figure 5a]. Although the calculated electronic energy barrier relative to the hydrated complex is modest, the formation of the covalent product during the first hydration step is not expected to be efficient under the experimental conditions. In particular, the association of a single water molecule involves a significant entropic penalty, as shown from the measured Δ*S*° of −27.0 cal mol^–1^K^–1^ and the calculated value at the M06–2*X*/6–311++G** level of 30.8 cal mol^–1^K^–1^ (Table 1). Consequently, the population of the monohydrated complex is expected to be limited in the gas phase, reducing the effective flux through the subsequent reaction pathway. Under such conditions, even a small internal barrier following complex formation may be sufficient to suppress observable product formation. Moreover, a single water molecule is unlikely to provide the extended hydrogen-bonding network required to efficiently stabilize the transition region associated with proton transfer and covalent bond formation. As a result, the reaction pathway identified for the monohydrated system may represent an energetically accessible but kinetically insignificant channel. In contrast, the experimental observation that covalent product formation becomes apparent with increasing water content suggests that higher degrees of microsolvation play a critical role in enabling the reaction. The presence of additional water molecules is expected to stabilize both the prereactive complex and the transition state through cooperative hydrogen bonding and proton-relay mechanisms, thereby lowering the effective free energy barrier to covalent bond formation. Such water-assisted pathways are well documented in gas phase ion–molecule intracluster reactions and provide a natural explanation for the experimentally observed dependence of product formation on the number of water molecules associating with the reactant ion.^16,24,25^

**Figure 7:**
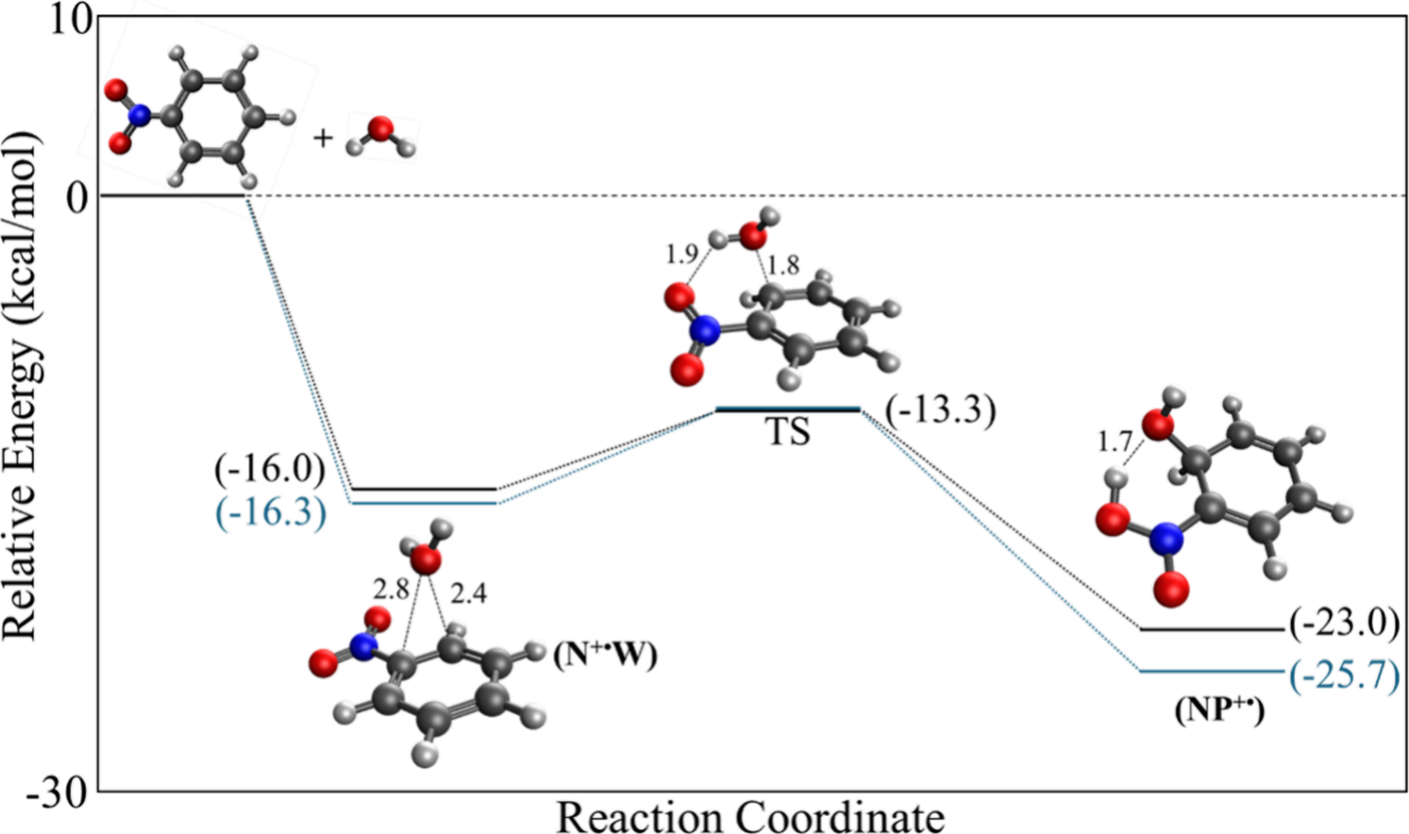
PES of the reaction of the nitrobenzene radical cation (C_6_H_5_NO_2_^+·^) with water toward the covalently bonded nitronic phenol-type radical cation (OH)­C_6_H_5_(NOOH)^·+^. The energies are calculated at the ϖB97M-V/6-311++G** (black) and the M06–2*X*/6–311++G** (blue) levels of theory.

To evaluate the entropic contributions to the isomerization of the noncovalent microhydrated nitrobenzene radical cation, we calculated the entropy values (S, cal mol^–1^K^–1^) for the species involved in the sequential hydration of the nitrobenzene radical cation C_6_H_5_NO_2_^·+^(H_2_O)_*n*_ with *n* = 1–3, and the covalently bonded ion C_6_H_7_NO_3_^·+^(H_2_O)_*n*_ with *n* = 1–5 at the M06–2*X*/6–311++G** level, and the results are listed in Table S1 (Supporting Information). Using the entropy data listed in Table S1, it can be shown that the reaction of the nitrobenzene radical cation with water to form the covalently bonded nitronic phenol-type radical cation, (OH)­C_6_H_5_(NOOH)^·+^ (**NP** ^**+·**^, Figure 5a), also involves an entropy barrier (Δ*S*°= −41.7 cal mol^–1^K^–1^) in addition to the identified energy barrier of 2.7 – 3.0 kcal/mol shown in Figure 7. Similarly, the reactions of the hydrated nitrobenzene radical cations, **NW** ^**+·**^ (Figure 4a); **NW**_**2**_^**+·**^ (Figure 4b); and **NW**_**3**_^**+·**^ (Figure 4c) with water to form the isomerized nitronic phenol-type radical cations **NP** ^**+·**^ **W** (Figure 5b); **NP** ^**+·**^ **W**_**2**_ (Figure 5c); and **NP** ^**+·**^ **W**_**3**_ (Figure 6a), respectively, involve significant loss of entropy (−37.4, −40.6 and −43.2 cal mol^–1^K^–1^, respectively). It should be noted that this entropy loss is larger than the typical entropy loss (∼30 cal mol^–1^K^–1^) associated with the noncovalent hydration of the nitrobenzene radical cation, as shown in Table 1.

Based on the experimental observations of sequential reversible association of three water molecules with the nitrobenzene radical cation and irreversible addition of water to the C_6_H_5_NO_2_^·+^(H_2_O)_3_ cluster [i.e., (N^·+^W_3_)], it is clear that the addition of fourth water molecule on (N^·+^W_3_) involves barrierless isomerization to the hydrated covalently bound nitrophenol-type radical cation [(HO)­C_6_H_5_(NOOH)]^·+^(H_2_O)_3_ [i.e., (NP^·+^W_3_)]. This irreversible transformation prevents the establishment of equilibrium between the third and fourth hydration steps of the nitrobenzene radical cation, consistent with the experimental observation. Therefore, the current experiments demonstrate that the C_6_H_5_NO_2_^·+^(H_2_O)_3_ cluster undergoes water-mediated isomerization involving a concerted proton transfer from the fourth water molecule to the N–O group of the nitrobenzene radical cation, accompanied by nucleophilic addition of the hydroxyl group to the ortho carbon, leading to the formation of the nitrophenol-type radical cation (NP^+·^) within the (NP^·+^W_3_) cluster. The irreversible isomerization of the (N^·+^W_3_) cluster [structure Figure 4c] to the (NP^·+^W_3_) cluster [structure Figure 6a] by the addition of the fourth water molecule is presented schematically in Figure 8. It is clear that the addition of the fourth water molecule on the (N^+·^W_3_) cluster is facilitated by a bridge of three water molecules in (N^+·^W_3_) [structure Figure 4c]. The ESP maps presented in Figure 8 show clearly that the enhanced electron density on the oxygen atom of the fourth water molecule facilitates the addition onto the electron-deficient region near the ortho carbon of the nitrobenzene ring simultaneously with a proton transfer to the oxygen atom of the NO_2_ group. Therefore, the water bridge provides a possible mechanism for the concerted proton transfer connecting the NO_2_ group with the nucleophilic addition of the hydroxyl group to the ortho carbon and resulting in the formation of a 6-membered ring structure (C–C–O–H–O–N–C) which could provide extra stability to the (NP^·+^W_3_) cluster [structure Figure 6a].

**Figure 8:**
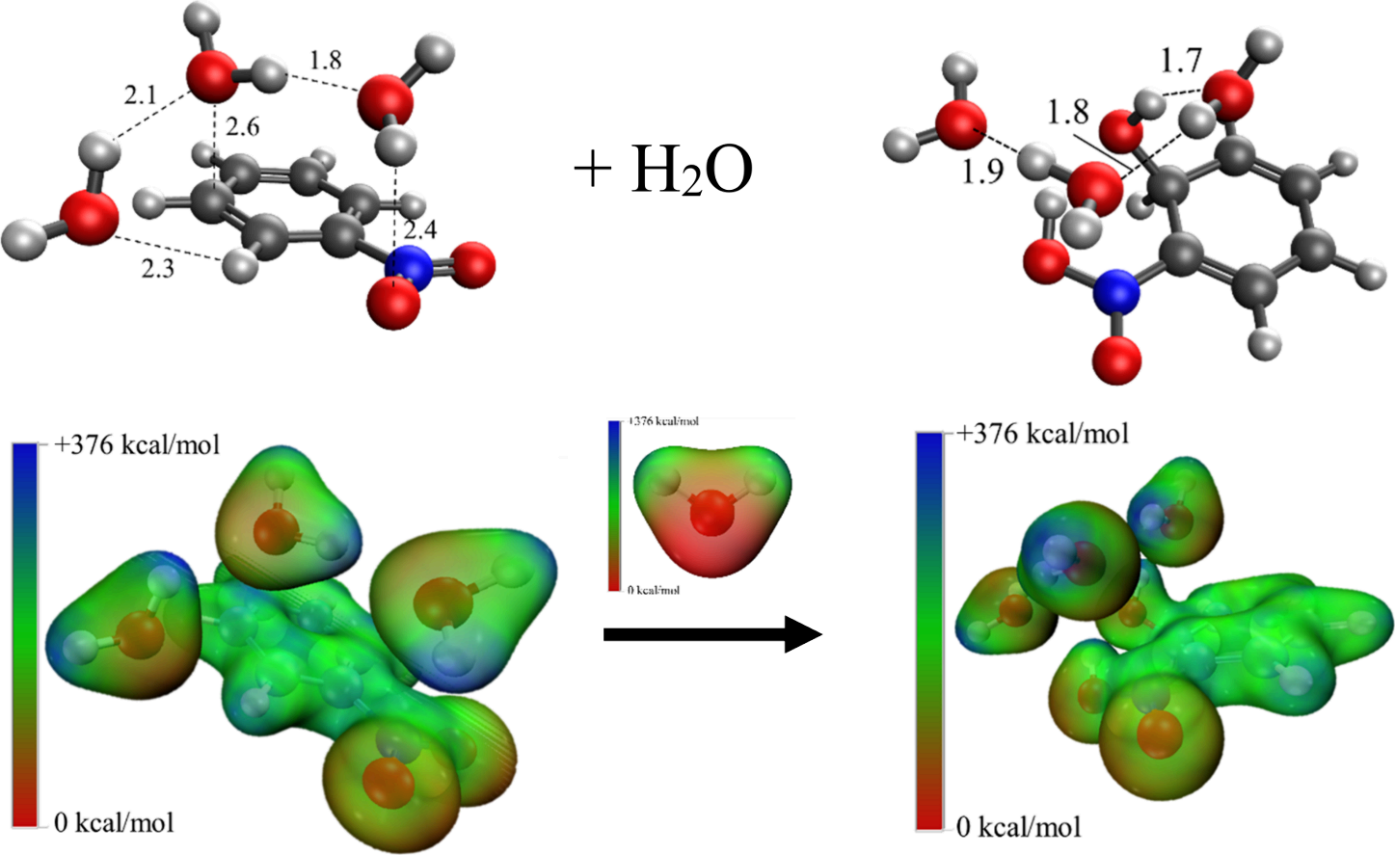
ESP representations of the irreversible transformation of nitrobenzene radical cation hydrated by three water molecules C_6_H_5_NO_2_^·+^(H_2_O)_3_ [i.e., (N^+·^W_3_), structure Figure 4c] to the hydrated nitrophenol-type radical cation hydrated by three water molecules (HO)­C_6_H_5_(NOOH)]^·+^(H_2_O)_3_, [i.e., (NP^+·^W_3_), structure Figure 6a], by addition of the fourth water molecule. Dark blue areas within the ESP maps indicate regions of electron deficiency or electropositive regions, while deep red areas represent enhanced electron density.

Figure 9 illustrates the overall reaction pathways suggested based on the calculated lowest energy structures and the corresponding −Δ*H*° values of the hydrated nitrobenzene radical cation clusters C_6_H_5_NO_2_^·+^(H_2_O)_*n*_ with *n* = 1–3 and the hydrated nitrophenol-type radical cation [(HO)­C_6_H_5_(NOOH)]^·+^(H_2_O)_*n*_ with *n* = 4–5. As shown in Figure 9, the reaction pathway between the nitrobenzene radical cation and water begins with the formation of a noncovalent hydrated complex, (N^+·^W), with a measured bonding enthalpy (−Δ*H*°) of 13.4 kcal/mol in excellent agreement with the calculated noncovalent interaction of 13.1 kcal/mol. Isomerization of (N^+·^W) [C_6_H_5_NO_2_^·+^(H_2_O)] to the more stable nitrophenol-type radical cation (NP^+·^) [(HO)­C_6_H_5_(NOOH)]^·+^ is prevented by an energy barrier and therefore it undergoes noncovalent association with water to form (N^+·^W_2_) with a measured bonding enthalpy of 12.7 kcal/mol, in excellent agreement with the calculated enthalpy change of 12.8 kcal/mol for structure (N^+·^W_2_). Although the barriers for isomerization of (N^+·^W_2_) to (NP^+·^W) and of (N^+·^W_3_) to (NP^+·^W_2_) vanish, the conversions to the (NP^+·^W) and (NP^+·^W_2_) structures remain kinetically insignificant, as suggested by the observation of reversible noncovalent association between (N^+·^W_2_) and (N^+·^W_3_), which results in a measured bonding enthalpy of 10.8 kcal/mol, again in excellent agreement with the calculated enthalpy change of 10.7 kcal/mol for structure (N^+·^W_3_). However, the observation of irreversible addition of water to the (N^·+^W_3_) cluster clearly indicates that the addition of the fourth water molecule onto (N^·+^W_3_) involves a barrierless isomerization to the hydrated covalently bound nitrophenol radical cation (NP^·+^W_3_). Interestingly, the experimental observation confirms the establishment of equilibrium reversible associations between (NP^·+^W_3_) ⇌ (NP^·+^W_4_) and between (NP^·+^W_4_) ⇌ (NP^·+^W_5_), resulting in measured bonding enthalpy of 11.7 and 12.1 kcal/mol, respectively, which are in good agreements with the calculated enthalpy changes of 11.9 and 11.4 kcal/mol for structures (NP^.+^W_4_) and (NP^.+^W_5_), respectively.

**Figure 9:**
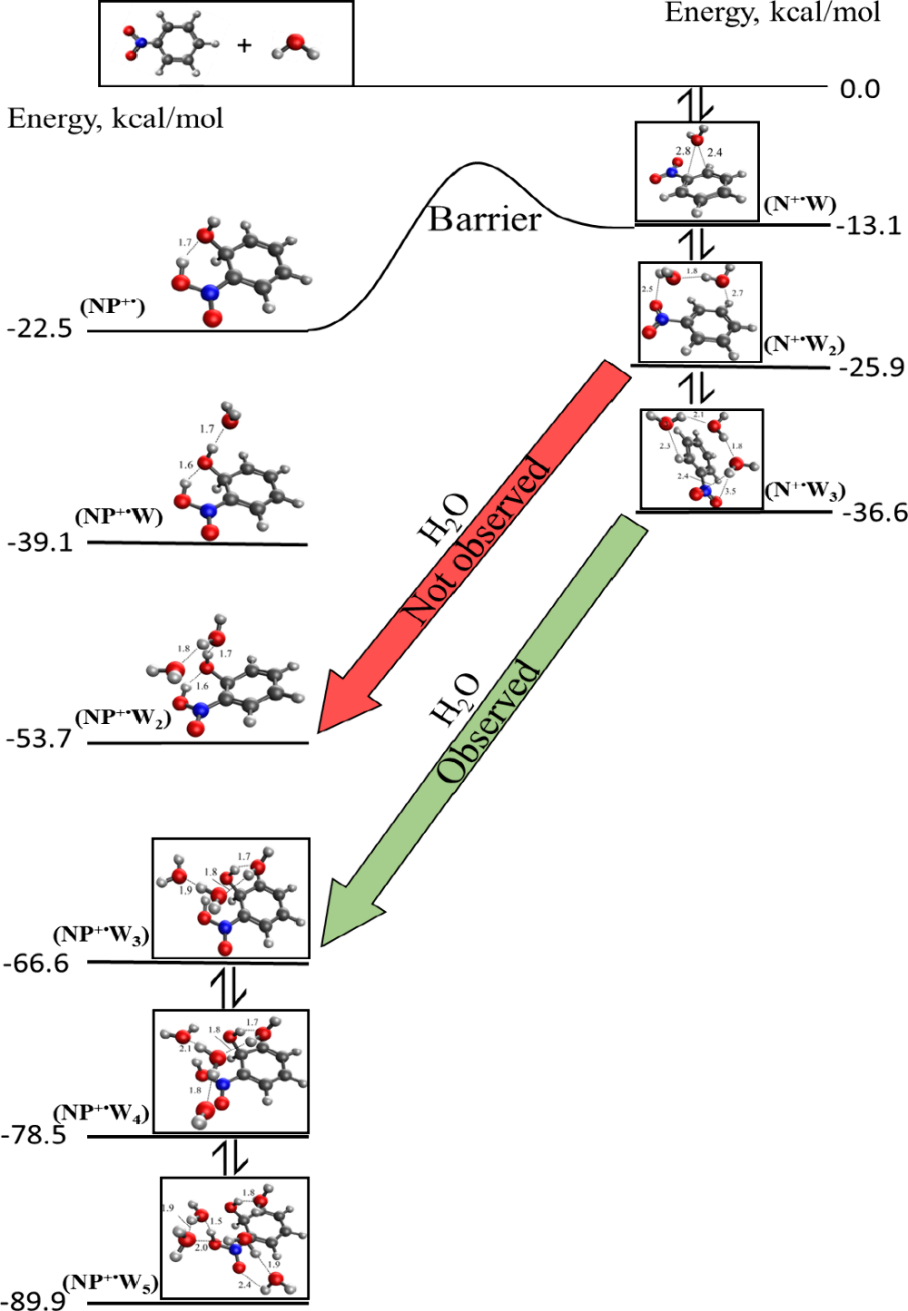
Reaction pathways for the sequential addition of water onto the nitrobenzene radical cation forming the hydrated clusters C_6_H_5_NO_2_^·+^(H_2_O)_*n*_, [N^+·^W_*n*_] with *n* = 1–3, followed by isomerization to generate the hydrated nitrophenol-type radical cation (HO)­C_6_H_5_(NOOH)]^·+^(H_2_O)_*n*_, and [NP^+·^W_*n*_] with *n* = 3–5. Listed values are Δ*H°* (kcal/mol) relative to the enthalpies of the starting reactants.

The formation of the hydrated nitronic phenol-type radical cation has significant implications for the production of gas-phase reactive hydrated organic nitrates (RONO_2_)^·+^(H_2_O)_*n*_ in atmospheric organic aerosols, which could contribute to air quality in a variety of environments.^26,27^ It is well-established that the gas-phase oxidation of volatile organic compounds such as nitrobenzene involves a variety of chemical pathways which could include the observed water-mediated isomerization mechanism of the C_6_H_5_NO_2_^·+^(H_2_O)_*n*_ clusters leading to the production of hydrated nitrophenol-type radical cation.^26,27^ These products can influence air quality through the production of organic aerosol particles in a variety of environments, including urban areas where higher concentrations of volatile organic nitrates are observed.^28^

## Conclusions and Outlook

The gas-phase stepwise hydration of the nitrobenzene radical cation with 1–6 water molecules has been investigated by means of ion mobility mass spectrometry and DFT calculations. The stepwise binding energies (Δ*H*°_*n*–1,*n*_) were determined by equilibrium measurements for C_6_H_5_NO_2_^·+^(H_2_O)_*n*_ with *n* = 1–3 as 13.4, 12.7, and 10.8 kcal/mol, respectively. DFT calculations indicate that the first three hydration steps (*n* = 1–3) are facilitated by noncovalent interactions of the associating water molecules with calculated enthalpy changes of 13.1, 12.8, and 10.7 kcal/mol, respectively, in excellent agreement with the experimentally determined Δ*H*° values. However, the fourth hydration step involves irreversible addition of water to the C_6_H_5_NO_2_^·+^(H_2_O)_3_ cluster, resulting in the formation of a covalently bonded ion hydrated by three water molecules [C_6_H_7_NO_3_^·+^(H_2_O)_3_]. This irreversible transformation prevents the establishment of equilibrium between the third and fourth hydration steps of the nitrobenzene radical cation, consistent with the experimental observation. Therefore, the current experiments demonstrate that the C_6_H_5_NO_2_^·+^(H_2_O)_3_ cluster undergoes water-mediated isomerization involving a concerted proton transfer from the fourth water molecule to the N–O group of the nitrobenzene radical cation, accompanied by nucleophilic addition of the hydroxyl group to the ortho carbon, leading to the formation of the nitrophenol-type radical cation within the hydrated clusters C_6_H_7_NO_3_^·+^(H_2_O)_*n*_ with *n* > 3. The formation of the hydrated nitronic phenol-type radical cation has significant implications for the production of gas-phase reactive hydrated organic nitrates (RONO_2_)^·+^(H_2_O)_*n*_ in atmospheric organic aerosols, which could contribute to air quality in a variety of environments.

## Supplementary Material



## Notes

Experimental setup of the mass-selected ion mobility system (Figure S1); reaction time profiles for the sequential addition of water onto the nitrobenzene radical cation C_6_H_5_NO_2_^·+^(H_2_O)_*n*_, with *n* = 1–3 and the hydrated covalently bonded ions C_6_H_7_NO_3_^·+^(H_2_O)_*n*_, with *n* = 3–4 (Figure S2); structures of the three lowest energy isomers of the C_6_H_5_NO_2_^+·^(H_2_O) cluster (Figure S3); structures of the three lowest energy isomers of the C_6_H_5_NO_2_^+·^(H_2_O)_2_ cluster (Figure S4); structures of the three lowest energy isomers of the C_6_H_5_NO_2_^+·^(H_2_O)_3_ cluster (Figure S5); and the calculated entropy values for the sequential hydration of the nitrobenzene radical cation C_6_H_5_NO_2_^·+^(H_2_O)_*n*_,with *n* = 1–3, and the covalently bonded ion C_6_H_7_NO_3_^·+^(H_2_O)_*n*_,with *n* = 1–5 (Table S1) (PDF)

## References

[1] Jeffrey, G. A. ; Saenger, W. Hydrogen Bonding in Biological Systems; Springer: Heidelberg, 1991.

[2] Noncovalent Forces, Scheiner, S. , Ed.; Springer International Publishing, 2015.

[3] Levy Y., Onuchic J. N. (2006). Water Mediation
in Protein Folding and Molecular Recognition. Annu. Rev. Biophys. Biomol. Struct..

[4] Swanson J. M. J., Maupin C. M., Chen H., Petersen M. K., Xu J., Wu Y., Voth G. A. (2007). Proton
Solvation and Transport in Aqueous and Biomolecular
Systems: Insights from Computer Simulations. J. Phys. Chem. B.

[5] Gudipati R. S., Allamandola L. J. (2006). Unusual Stability of Polycyclic Aromatic Hydrocarbon
Radical Cations in Amorphous Water Ices up to 120 K: Astronomical
Implications. Astrophys. J..

[6] Menor-Salvan C., Marin-Yaseli M. R. (2012). Prebiotic
Chemistry in Eutectic Solutions at the Water–Ice
Matrix. Chem. Soc. Rev..

[7] Ibrahim Y., Alsharaeh E., Dias K., Meot-Ner M., El-Shall M. S. (2004). Stepwise
Hydration and Multibody Deprotonation with Steep Negative Temperature
Dependence in the Benzene^+·^Water System. J. Am. Chem. Soc..

[8] Ibrahim Y. M., Meot-Ner M., Alshraeh E. H., El-Shall M. S., Scheiner S. (2005). Stepwise Hydration
of Ionized Aromatics. Energies, Structures of the Hydrated Benzene
Cation, and the Mechanism of Deprotonation Reactions. J. Am. Chem. Soc..

[9] Ibrahim Y., Mabrouki R., Meot-Ner M., El-Shall M. S. (2007). Hydrogen Bonding
Interactions of Pyridine^·+^ with Water: Stepwise Solvation
of Distonic Cations. J. Phys. Chem. A.

[10] Momoh P. O., El-Shall M. S. (2008). Gas Phase Hydration
of Organic Ions. Phys. Chem. Chem. Phys..

[11] Momoh P. O., Hamid A. M., Abrash S. A., El-Shall M. S. (2011). Structure and Hydration
of the C_4_H_4_
^·+^ Ion formed by
Electron Impact Ionization of Acetylene Clusters. J. Chem. Phys..

[12] Hamid A. M., Sharma P., El-Shall M. S., Hilal R., Elroby S., Aziz S. G., Alyoubi A. O. (2013). Hydration
of the Pyrimidine Radical
Cation and Stepwise Solvation of Protonated Pyrimidine with Water,
Methanol and Acetonitrile. J. Chem. Phys..

[13] Haupert L. J., Wenthold P. G. (2013). Hydration Energies
of Aromatic Ions in the Gas Phase. J. Phys.
Chem. A.

[14] Attah I. K., Platt S. P., Mautner M., El-Shall M. S., Aziz S. G., Alyoubi A. O. (2014). Hydrogen Bonding
of the Naphthalene Radical Cation
to Water and Methanol and Attachment of the Naphthalene Ion to Extended
Hydrogen Bonding Chains. Chem. Phys. Lett..

[15] El-Shall, M. S. ; Attah, I. K. ; Platt, S. P. Noncovalent Interactions of Organic Ions with Polar Molecules in the Gas Phase, Chapter 15. In Noncovalent Forces, Scheiner, S. , Ed.; Springer, 2015; pp 443–469.

[16] Mason K. A., Pearcy A. C., Christensen Z. A., Attah I. K., Meot-Ner M., El-Shall M. S. (2022). Water-Assisted Proton Transfer in the Sequential Hydration
of Benzonitrile Radical Cation C_6_H_5_CN^·+^(H_2_O)_
*n*
_: Transition to Hydrated
Distonic Cation ^·^C_6_H_4_CNH^+^(H_2_O)_
*n*
_ with *n* ≥ 4. J. Am. Chem. Soc..

[17] Soliman A. R., Hamid A. M., Abrash S. A., El-Shall M. S. (2012). Unconventional
Ionic
Hydrogen Bonds: CH^+^···π (C≡C)
Binding Energies and Structures of Benzene^+^(Acetylene)_1–4_ Clusters. Chem. Phys. Lett..

[18] Meot-Ner M. (2012). Update 1 of:
Strong Ionic Hydrogen Bonds. Chem. Rev..

[19] Sutton P., Saunier J., Mason K. A., Pearcy A. C., Lao K. U., El-Shall M. S. (2024). Formation of Complex
Organics by Covalent and Non-covalent
Interactions of the Sequential Reactions of 1–4 Acrylonitrile
Molecules with the Benzonitrile Radical Cation. Phys. Chem. Chem. Phys..

[20] Sutton P., Saunier J., Lao K. U., El-Shall M. S. (2024). Sequential Reactions
of Acetylene with the Benzonitrile Radical Cation: New Insights into
Structures and Rate Coefficients of the Covalent Ion Products. J. Phys. Chem. Lett..

[21] Frisch, M. J. ; Trucks, G. W. ; Schlegel, H. B. ; Scuseria, G. E. ; Robb, M. A. ; Cheeseman, J. R. ; Scalmani, G. ; Barone, V. ; Petersson, G. A. ; Nakatsuji, H. ; Li, X. ; Caricato, M. ; Marenich, A. V. ; Bloino, J. ; Janesko, B. G. ; Gomperts, R. ; Mennucci, B. ; Hratchian, H. P. ; Ortiz, J. V. ; Izmaylov, A. F. ; Sonnenberg, J. L. ; Williams-Young, D. ; Ding, F. ; Lipparini, F. ; Egidi, F. ; Goings, J. ; Peng, B. ; Petrone, A. ; Henderson, T. ; Ranasinghe, D. ; Zakrzewski, V. G. ; Gao, J. ; Rega, N. ; Zheng, G. ; Liang, W. ; Hada, M. ; Ehara, M. ; Toyota, K. ; Fukuda, R. ; Hasegawa, J. ; Ishida, M. ; Nakajima, T. ; Honda, Y. ; Kitao, O. ; Nakai, H. ; Vreven, T. ; Throssell, K. ; Montgomery, J. A., Jr. ; Peralta, J. E. ; Ogliaro, F. ; Bearpark, M. J. ; Heyd, J. J. ; Brothers, E. N. ; Kudin, K. N. ; Staroverov, V. N. ; Keith, T. A. ; Kobayashi, R. ; Normand, J. ; Raghavachari, K. ; Rendell, A. P. ; Burant, J. C. ; Iyengar, S. S. ; Tomasi, J. ; Cossi, M. ; Millam, J. M. ; Klene, M. ; Adamo, C. ; Cammi, R. ; Ochterski, J. W. ; Martin, R. L. ; Morokuma, K. ; Farkas, O. ; Foresman, J. B. ; Fox, D. J. Gaussian 16, Revision C.01; Gaussian, Inc.: Wallingford CT, 2016.

[22] Sharada S. M., Zimmerman P. M., Bell A. T., Head-Gordon M. (2012). Automated
Transition State Searches without Evaluating the Hessian. J. Chem. Theory Comput..

[23] Epifanovsky E., Gilbert A. T. B., Feng X. (2021). Software for the frontiers
of quantum chemistry: An overview of developments in the Q-Chem 5
package. J. Chem. Phys..

[24] Rana A., Harville P. A., Khuu T., LaCour R. A., Head-Gordon T., Johnson M. A. (2025). The Onset for Vibrationally
Induced, Intramolecular
Proton Transfer at Three Water Molecules in Microhydrated 4-Aminobenzoic
Acid. J. Phys. Chem. Lett..

[25] Rana A., Harville P. A., Khuu T., Johnson M. A. (2025). Microcanonical Kinetics
of Water-mediated Proton Transfer in Microhydrated 4-Aminobenzoic
Acid. Science.

[26] Hettiarachchi E., Grassian V. H. (2024). Heterogeneous Reactions
of Phenol on Different Components
of Mineral Dust Aerosol: Formation of Oxidized Organic and Nitro-Phenolic
Compounds. ACS ES&T Air.

[27] Crounse J. D., Nielsen L. B., Jørgensen S., Kjaergaard H. G., Wennberg P. O. (2013). Autoxidation of Organic Compounds
in the Atmosphere. J. Phys. Chem. Lett..

[28] Lee B. H., Mohr C., Lopez-Hilfiker F. D., Lutz A., Hallquist M., Lee L., Romer P., Cohen R. C., Iyer S., Kurtén T., Hu W., Day D. A., Campuzano-Jost P., Jimenez J. L., Xu L., Ng N. L., Guo H., Weber R. J., Wild R. J., Brown S. S., Koss A., de Gouw J., Olson K., Goldstein A. L., Seco R., Kim S., McAvey K., Shepson P. B., Starn T., Baumann K., Edgerton E. S., Liu J., Shilling J. E., Miller D. O., Brune W., Schobesberger S., D’Ambro E. L., Thornton J. A. (2016). Highly Functionalized Organic Nitrates
in the Southeast United States: Contribution to Secondary Organic
Aerosol and Reactive Nitrogen Budgets. Proc.
Natl. Acad. Sci. U. S. A..

