# Supplementary material for: Water-Mediated Isomerization in the Stepwise Hydration of the Nitrobenzene Radical Cation with 1–6 Water Molecules
Source: J Phys Chem A. 2026 Mar 23;130(15):2968–78. doi: 10.1021/acs.jpca.5c08557 (PMC13093476; doi:10.1021/acs.jpca.5c08557)
Supplement: Supplementary file 1 [file jp5c08557_si_001.pdf]

Water-mediated Isomerization in the Stepwise Hydration of the Nitrobenzene Radical Cation with 1-6 Water Molecules

Zachary A. Christensen, John P. Saunier, Kyle A. Mason, Ka Un Lao, and M. Samy El-Shall*

Department of Chemistry, Virginia Commonwealth University,
Richmond, VA 23284-2006, United States

Supporting Information

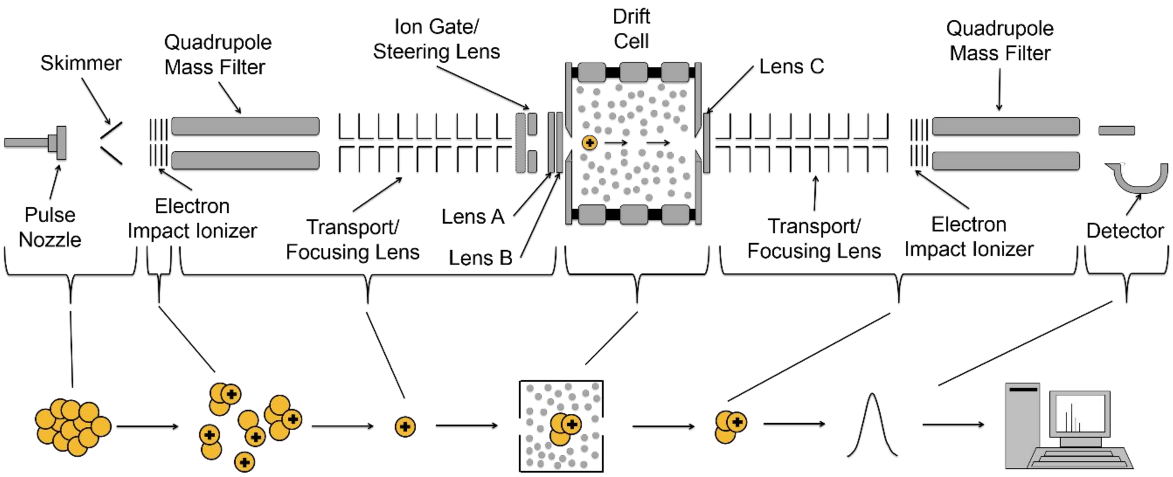

Figure S1. Experimental Set-up of the Mass-Selected Ion Mobility System.

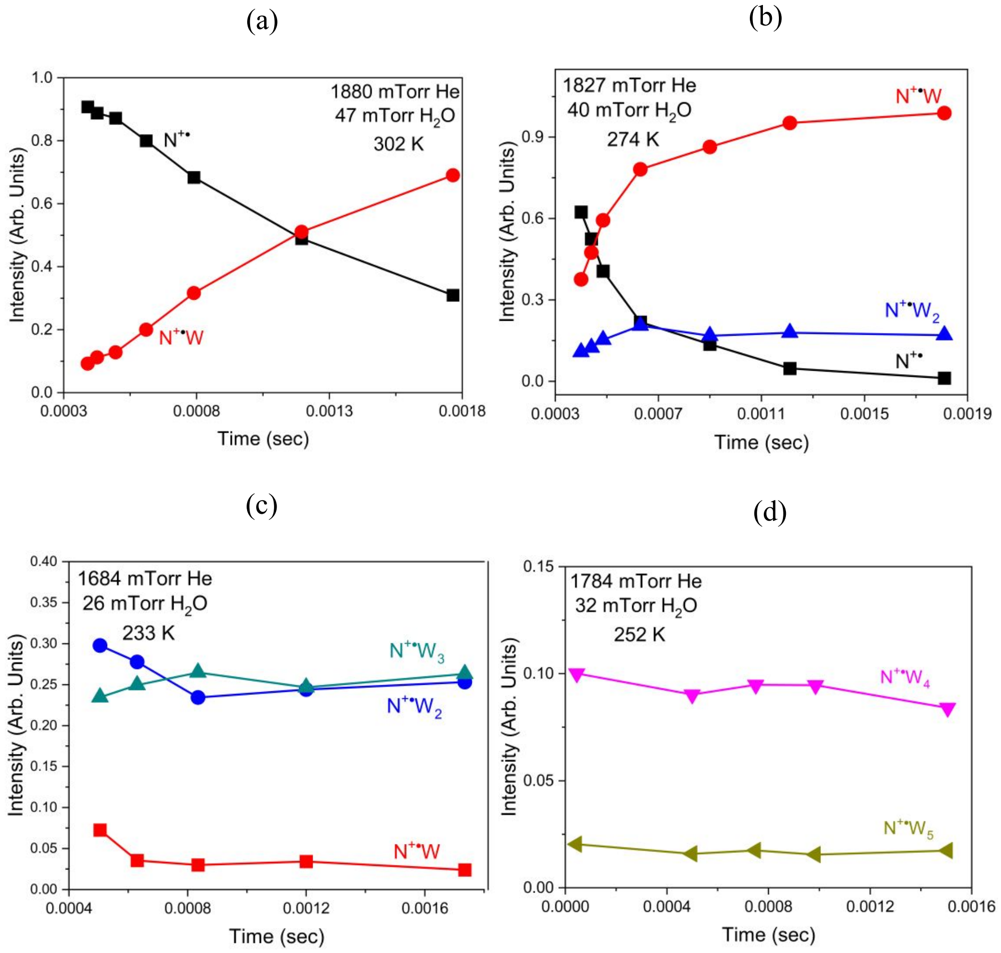

Figure S2. Time profiles for the reaction of nitrobenzene radical cation ($N^{+\bullet}$, black line) with water (W) leading to the first ($N^{+\bullet}W$, red line), second ($N^{+\bullet}W_2$, blue line), third ($N^{+\bullet}W_3$, green line), fourth ($N^{+\bullet}W_4$, magenta line), and fifth ($N^{+\bullet}W_5$, dark yellow line) hydration products at the indicated pressures and temperatures of: (a) 302 K, (b) 274 K, (c) 233 K, and (d) 252 K. $N^{+\bullet}W_4$ and $N^{+\bullet}W_5$ represent the time profiles of the hydrated covalently-bonded ions $C_6H_7NO_3^{\bullet+}(H_2O)_3$ and $C_6H_7NO_3^{\bullet+}(H_2O)_4$, respectively.

(a)	(b)	(c)
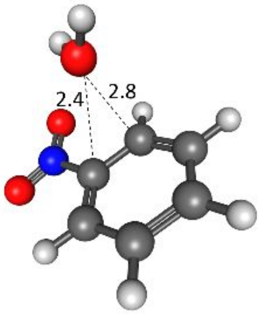 **$(N^{+\bullet}W)$** -*ΔH*° = 13.1 kcal/mol	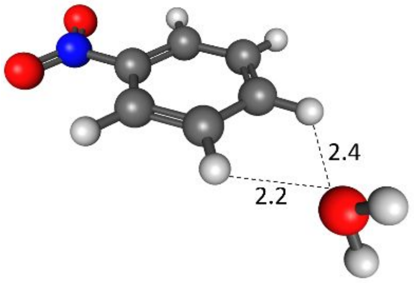 **$(N^{+\bullet}W)$** -*ΔH*° = 10.2 kcal/mol	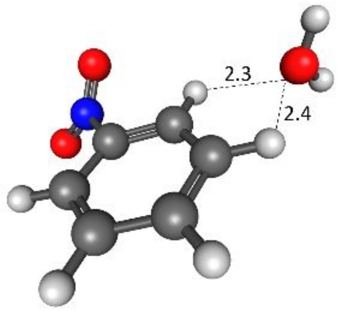 **$(N^{+\bullet}W)$** -*ΔH*° = 8.0 kcal/mol

Figure S3. Structures and binding enthalpies (*ΔH*°, kcal/mol) calculated at the M06-2X/6-311++G** level of theory for the three lowest energy isomers: **(a)** -*ΔH*° = 13.1 kcal/mol, **(b)** -*ΔH*° = 10.2 kcal/mol, and **(c)** -*ΔH*° = 8.0 kcal/mol of the $C_6H_5NO_2^{+}(H_2O)$ cluster. The distances shown on the dotted lines are in angstroms.

(a)	(b)	(c)
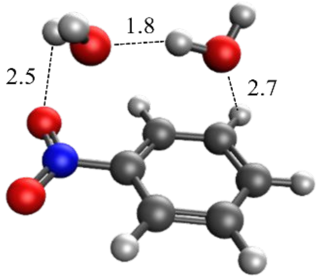 **$(N^{+\bullet}W_2)$** -*ΔH*° = 12.8 kcal/mol	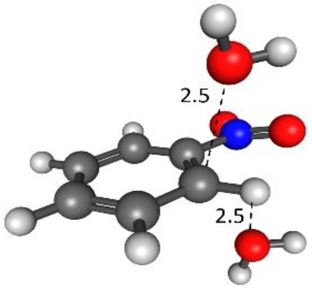 **$(N^{+\bullet}W_2)$** -*ΔH*° = 12.4 kcal/mol	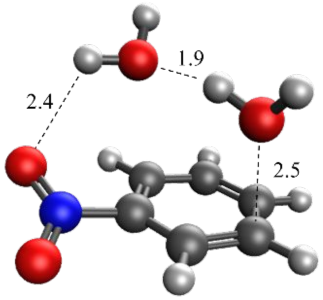 **$(N^{+\bullet}W_2)$** -*ΔH*° = 11.8 kcal/mol

Figure S4. Structures and binding enthalpies (*ΔH*°, kcal/mol) calculated at the M06-2X/6-311++G** level of theory for the three lowest energy isomers: **(a)** -*ΔH*° = 12.8 kcal/mol, **(b)** -*ΔH*° = 12.4 kcal/mol, and **(c)** -*ΔH*° = 11.8 kcal/mol of the $C_6H_5NO_2^{+}(H_2O)_2$ cluster. Distances are reported in angstroms.

(a)	(b)	(c)
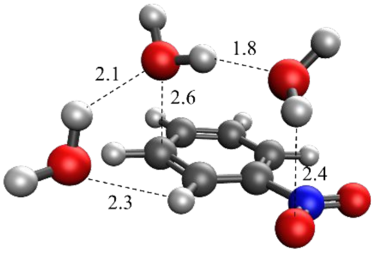<br>**($N^{+\bullet}W_3$)**<br>-Δ*H* = 10.7 kcal mol^{-1}	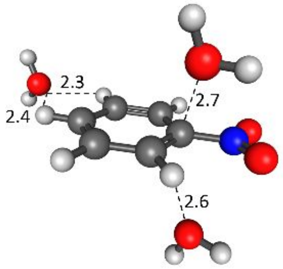<br>**($N^{+\bullet}W_3$)**<br>-Δ*H* = 8.8 kcal mol^{-1}	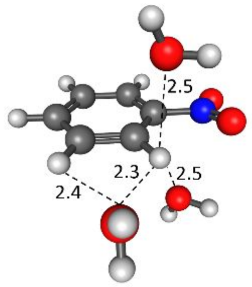<br>**($N^{+\bullet}W_3$)**<br>-Δ*H* = 8.3 kcal mol^{-1}

Figure S5. Structures and binding enthalpies (ΔH, kcal/mol) calculated at the M06-2X/6-311++G** level of theory for the three lowest energy isomers: **(a)** -*ΔH*° = 10.7 kcal/mol, **(b)** -*ΔH*° = 8.8 kcal/mol, and **(c)** -*ΔH*° = 8.3 kcal/mol of the $C_6H_5NO_2^{+\cdot}(H_2O)_3$ cluster. Distances are reported in angstroms.

Table S1

Calculated entropy values (S, cal mol^{-1}K^{-1}) for the sequential hydration of the nitrobenzene radical cation $C_6H_5NO_2^{\bullet+}(H_2O)_n$ with n = 1-3, and the covalently bonded ion $C_6H_7NO_3^{\bullet+}(H_2O)_n$ with n = 1-5 at the M06-2X/6-311++G** level.

Structure	Figure	S (cal mol^{-1}K^{-1})
H_2O		45.1
$C_6H_5NO_2^{\bullet+}$		87.5
$C_6H_5NO_2^{\bullet+}(H_2O)$ **($N^{+\bullet}W$)**	4(a)	101.7
$C_6H_5NO_2^{\bullet+}(H_2O)_2$ **($N^{+\bullet}W_2$)**	4(b)	116.0
$C_6H_5NO_2^{\bullet+}(H_2O)_3$ **($N^{+\bullet}W_3$)**	4(c)	130.4
$C_6H_7NO_3^{\bullet+}$ **($NP^{+\bullet}$)**	5(a)	90.9
$C_6H_7NO_3^{\bullet+}(H_2O)$ **($NP^{+\bullet}W$)**	5(b)	109.4
$C_6H_7NO_3^{\bullet+}(H_2O)_2$ **($NP^{+\bullet}W_2$)**	5(c)	120.5
$C_6H_7NO_3^{\bullet+}(H_2O)_3$ **($NP^{+\bullet}W_3$)**	6(a)	132.3
$C_6H_7NO_3^{\bullet+}(H_2O)_4$ **($NP^{+\bullet}W_4$)**	6(b)	146.0
$C_6H_7NO_3^{\bullet+}(H_2O)_5$ **($NP^{+\bullet}W_5$)**	6(c)	159.2
